# Machine Learning and Shapley Additive Explanations Value Integration for Predicting the Prognostic of Anti-N-Methyl-D-Aspartate Receptor Encephalitis: Model Development and Evaluation Study

**DOI:** 10.2196/75020

**Published:** 2025-09-22

**Authors:** Jia Wang, Haotian Wu, Han Cai, YingXiang Wang, Jian Gu

**Affiliations:** 1 Department of Gynecology, Third Affiliated Hospital of Sun Yat-sen University, 600 Tianhe Road, Tianhe District, Guangdong Province, Guangzhou, China, 86 85252237; 2 Department of Neurology, Third Affiliated Hospital of Sun Yat-sen University, Guangzhou, China

**Keywords:** anti-N-methyl-D-aspartate receptor (NMDAR) encephalitis, machine learning, SHAP, prognosis, prediction

## Abstract

**Background:**

Anti-N-methyl-D-aspartate receptor (NMDAR) encephalitis is a rare disease with no accurate prognostic tools to predict the prognosis of patients.

**Objective:**

This study aims to develop an interpretable machine learning model using real-world clinical data to guide personalized therapeutic strategies.

**Methods:**

This retrospective cohort study analyzed 140 patients with NMDAR encephalitis treated at the Third Affiliated Hospital of Sun Yat-sen University (2015‐2024). Feature selection was done using recursive feature elimination. The model was constructed by 3 machine learning algorithms: decision tree, random forest (RF), and extreme gradient boosting. Mean squared error, root-mean-squared error, *R*² (coefficient of determination), mean absolute error, and mean absolute percentage error were used to evaluate the model performance. Finally, the optimal model was interpreted via Shapley Additive Explanations (SHAP) and deployed as a web application using the Flask framework.

**Results:**

The median age of patients with anti-NMDAR encephalitis was 23 (IQR 18-31.8) years. The median Clinical Evaluation Scale for Autoimmune Encephalitis score at acute onset was 11 (IQR 6-16). After preprocessing, 20 features, including 4 demographic characteristics, 3 clinical characteristics, 11 laboratory parameters, and 2 neuroimaging characteristics, were selected. The RF demonstrated superior accuracy in predicting the prognosis (mean squared error=11.01; root-mean-squared error=3.32; *R*²=0.71; mean absolute error=2.49; mean absolute percentage error=0.48). SHAP analysis identified admission to the intensive care unit (mean |SHAP value|=1.65), initial symptoms-memory deficits (0.69), and uric acid (0.53) as the most important prognostic predictors.

**Conclusions:**

We developed and validated an interpretable RF-based prognostic model for NMDAR encephalitis. The web-deployed tool enables real-time risk stratification, facilitating clinical decision-making and personalized therapeutic interventions for clinicians.

## Introduction

Anti-N-methyl-D-aspartate receptor (NMDAR) encephalitis represents more than 80% of autoimmune encephalitis (AE) cases. Its pathogenesis is closely linked to synaptic plasticity impairment mediated by autoantibodies targeting the NMDAR GluN1 subunit [1,2]. These antibodies induce limbic system dysfunction and characteristic neuropsychiatric manifestations [3]. While first-line immunotherapy has markedly improved survival rates, up to 30%‐47% of patients continue to experience cognitive deficits, psychiatric symptoms, or functional disabilities [4-6]. Consequently, the development of a dynamic prognostic evaluation system is critical for guiding individualized treatment strategies.

Since the initial report of NMDAR encephalitis in 2007, researchers have investigated the prognostic value of various biomarkers. Current evidence indicates that cerebrospinal fluid antibody titers, extreme delta brush on electroencephalogram, and acute-phase inflammatory markers may correlate with disease severity [5,7-9]. However, single-dimensional biomarkers exhibit insufficient predictive power. Currently, there is a lack of integrated multidimensional data for constructing models to predict patient outcomes. Existing prediction models [10-12] predominantly rely on the modified Rankin Scale [13] and are limited in assessing the cognitive-mental dimension of NMDAR encephalitis. Moreover, traditional logistic regression approaches fail to adequately analyze complex nonlinear interactions among predictors, thereby reducing their clinical applicability.

Machine learning (ML) offers transformative potential for integrating and analyzing complex clinical data. In contrast to traditional statistical methods, ML algorithms can identify intricate feature interactions and enhance predictive performance in small-sample rare disease cohorts [14]. This capability has been successfully demonstrated in neurodegenerative diseases and tumor prognosis prediction by enhancing the feature representation of small-sample datasets. However, no study has systematically applied ML for prognostic prediction in NMDAR encephalitis.

The objective of this study is to integrate clinical features, neuroimaging findings, serological markers, and Clinical Evaluation Scale for Autoimmune Encephalitis (CASE) [15] scores to construct a prognostic prediction model for NMDAR encephalitis based on ML and identify early prognostic signatures through Shapley Additive Explanations (SHAP)–driven feature interpretability. Furthermore, a web-based clinical tool was developed to deploy the model, enabling clinicians to use it directly for prognostic predictions and to plan interventions (eg, escalation to second-line therapy or multiple immune combinations within a critical 4-week postsymptom window).

## Methods

### Study Design

Data for this retrospective study were collected from the Third Affiliated Hospital of Sun Yat-sen University from 2015 to 2024. This study was conducted in accordance with the Transparent Reporting of a Multivariable Prediction Model for Individual Prognosis or Diagnosis (TRIPOD) guidelines [16]. Informed consent was obtained from all participants. This study was approved by the ethics committee of the Third Affiliated Hospital of Sun Yat-sen University (II2024-370-01). This study comprised 3 phases: (1) cohort selection, (2) variable optimization and model development, and (3) validation and clinical translation tools.

### Study Population

All patients underwent comprehensive evaluations, including neuroimaging, electroencephalogram, cerebrospinal fluid (CSF) analysis, and tumor screening.

### Eligibility Criteria

The inclusion and exclusion criteria are listed in Textbox 1.

Textbox 1. **Inclusion and exclusion criteria.**

**Inclusion criteria**

- Age ≥10 years at disease onset.
- Diagnosis of anti-N-methyl-D-aspartate receptor encephalitis based on established criteria.
- Diagnosis before immunotherapy.

**Exclusion criteria**

- Concurrent central nervous system neoplasms or noninfectious inflammatory disorders.
- Documented central nervous system infections (bacterial, fungal, parasitic, or tuberculous).
- Secondary septic encephalopathy or systemic inflammatory response syndrome–associated encephalopathy.
- Coexisting serum or cerebrospinal fluid autoantibodies associated with other autoimmune encephalitides.
- Incomplete clinical documentation.

### Data Collection

Data were extracted from electronic medical records. Standardized variables included the following:

1. Demographics: age at disease onset, gender, occupation, and BMI.
2. Clinical characteristics: (1) prodromal symptoms (fever, headache, and dizziness) and (2) initial symptoms (psychiatric abnormalities, seizures, altered consciousness, and memory deficits).
3. Critical care interventions: intensive care unit (ICU) admission and mechanical ventilation requirement.
4. Laboratory parameters: CSF or serum anti-NMDAR antibody titers. CSF samples were analyzed using a cell-based assay by a trained specialist in a laboratory with experience in interpreting these assays. (1) Sex hormone tests include luteinizing hormone, follicle-stimulating hormone, estradiol, progestogen, and testosterone. (2) Hematological and biochemical analyses: white blood cell count, neutrophils, lymphocytes, monocytes, uric acid (UA), erythrocyte sedimentation rate, C-reactive protein, and high-density lipoprotein cholesterol.
5. Neuroimaging: Baseline magnetic resonance imaging (MRI) or electroencephalogram findings, including lesion quantity, and MRI. Analysis was performed by a senior neuroradiologist and neurologist using 1.5/3.0 Tesla scanners with T1-weighted, T2-weighted, and FLAIR sequences. All electroencephalogram data were recorded according to the international 10‐20 system using an electroencephalogram Digi Track (ELMIKO) with 19 electrodes. Tumor details (if applicable) included tumor size (mean of longitudinal and transverse diameters on imaging) and number of tumors.

### Evaluation Prognosis and Definitions

Outcome metric and clinical severity were quantified using the CASE scale, a validated 9-domain instrument assessing (seizures, memory deficits, psychiatric symptoms, consciousness, speech, motor or dystonia, gait or ataxia, brainstem dysfunction, muscle strength; total score: 0‐27). Prognostic stratification was defined as favorable: 0‐4 (minimal residual symptoms), moderate: 5‐9 (partial functional impairment), and poor: 10‐27 (severe disability). Lower CASE scores at follow-up indicated improved functional recovery.

### Data Preprocessing and Feature Engineering

Categorical variables were coded by labels’ encoding, while continuous variables were standardized using the Standard Scaler to mitigate feature scale bias. From an initial pool of 47 candidate predictors (selected via systematic literature review and clinical consensus), recursive feature elimination (RFE) with random forest (RF) (500 trees, Gini impurity) was applied. Features exceeding the importance threshold (*P*>.01) were retained. The threshold of 0.01 was empirically validated as optimal for maximizing cross-validation accuracy. This value consistently yielded peak performance in predicting prognosis across all candidate models (decision tree [DT], RF, extreme gradient boosting [XGBoost]), balancing feature retention with model generalizability. To rigorously mitigate randomness in RFE with a cross-validation workflow, we implemented a dual-strategy approach: (1) stratified cross-validation. Instead of relying on a single random train-test split, we used stratified 5-fold CV throughout the RFE process. (2) To test the stability of the selected feature set, we repeated the RFE with a cross-validation process multiple times with different random seeds (random_state=42). We then analyzed the frequency with which each feature was selected. Features that consistently appeared across multiple runs were considered more robust and were prioritized.

### Model Development and Validation

The dataset was strategically partitioned through random stratified sampling into a training set (80%, n=112) and a validation set (20%, n=28). The training set was exclusively used for model development, while the validation set served as an independent cohort for performance evaluation. The 3 ML algorithms, including RF, XGBoost, and DT, were used to construct the prediction model of NMDAR. Benchmarking is an important way to systematically evaluate and compare the performance of ML models. This process involves evaluating multiple models on standardized datasets and using consistent evaluation metrics to ensure fair comparisons. In this study, model performance was assessed using mean squared error (MSE), root-mean-squared error (RMSE), coefficient of determination (*R*²), mean absolute error, and mean absolute percentage error. The optimal model was selected based on minimal RMSE in validation. A 5-fold cross-validation was used to verify the stability of the model (Table 1).

**Table 1.** Five-fold cross-validation of the model.

Number of folds	MSE^a^	RMSE^b^	*R*²^c^	MAE^d^	MAPE^e^
1	10.52	3.24	0.73	2.41	0.46
2	11.38	3.37	0.69	2.57	0.5
3	10.79	3.28	0.72	2.46	0.47
4	11.62	3.41	0.68	2.63	0.51
5	10.95	3.31	0.71	2.49	0.48
Average	11.052	3.322	0.706	2.512	0.484

a MSE: mean square error.

b RMSE: root-mean-squared error.

c R²: coefficient of determination.

d MAE: mean absolute error.

e MAPE: mean absolute percentage error.

### Model Interpretation and Network Application

The ML has difficulty explaining the contribution of each feature due to its black-box principle. Interpretability denotes elucidating how ML models produce results. To elucidate feature contributions, SHAP values were used to quantify and interpret feature importance in the best-performing model. The algorithm provides a measure of feature importance across the model. Local interpretability was visualized via force plots, while global patterns were assessed through mean |SHAP values|.

### Network Application

Network application was deployed using Flask 2.3.2 backend with REST API, dynamic input forms aligned with selected predictors, real-time SHAP plot generation, and risk stratification output (probability scores with 95% CIs). This tool operates independently from any model and uses input predictor variables for result interpretation while enabling predictions for individual cases.

### Statistical Analysis

The Shapiro–Wilk normality test was performed to assess the data normality. Continuous variables are reported as medians with interquartile ranges (IQRs) for skewed distributed variables. Categorical variables are reported as frequency and percentage.

All statistical analyses were performed using IBM SPSS Statistics 22 (SPSS Inc). The predictive model construction and graphical representations were implemented using Python V3.7 (Python Software Foundation) and Prism 10.0 (GraphPad Software), respectively.

### Ethical Considerations

The Ethics Committee of the Third Affiliated Hospital, Sun Yat-sen University (II2024-370–01) gave ethical approval on December 30, 2024. Informed consent was waived by the committee, as the study involved retrospective analysis of anonymized data and did not include any identifiable personal information. All procedures were conducted in accordance with the ethical standards of the institutional and national research committees and with the principles outlined in the Declaration of Helsinki. We ensured that the privacy and confidentiality of all participants were strictly maintained; data were fully anonymized before analysis, and no individual-level identifiers were collected, stored, or reported. No compensation was provided to participants, as the study did not involve direct interaction with human participants.

## Results

**Figure 1.**
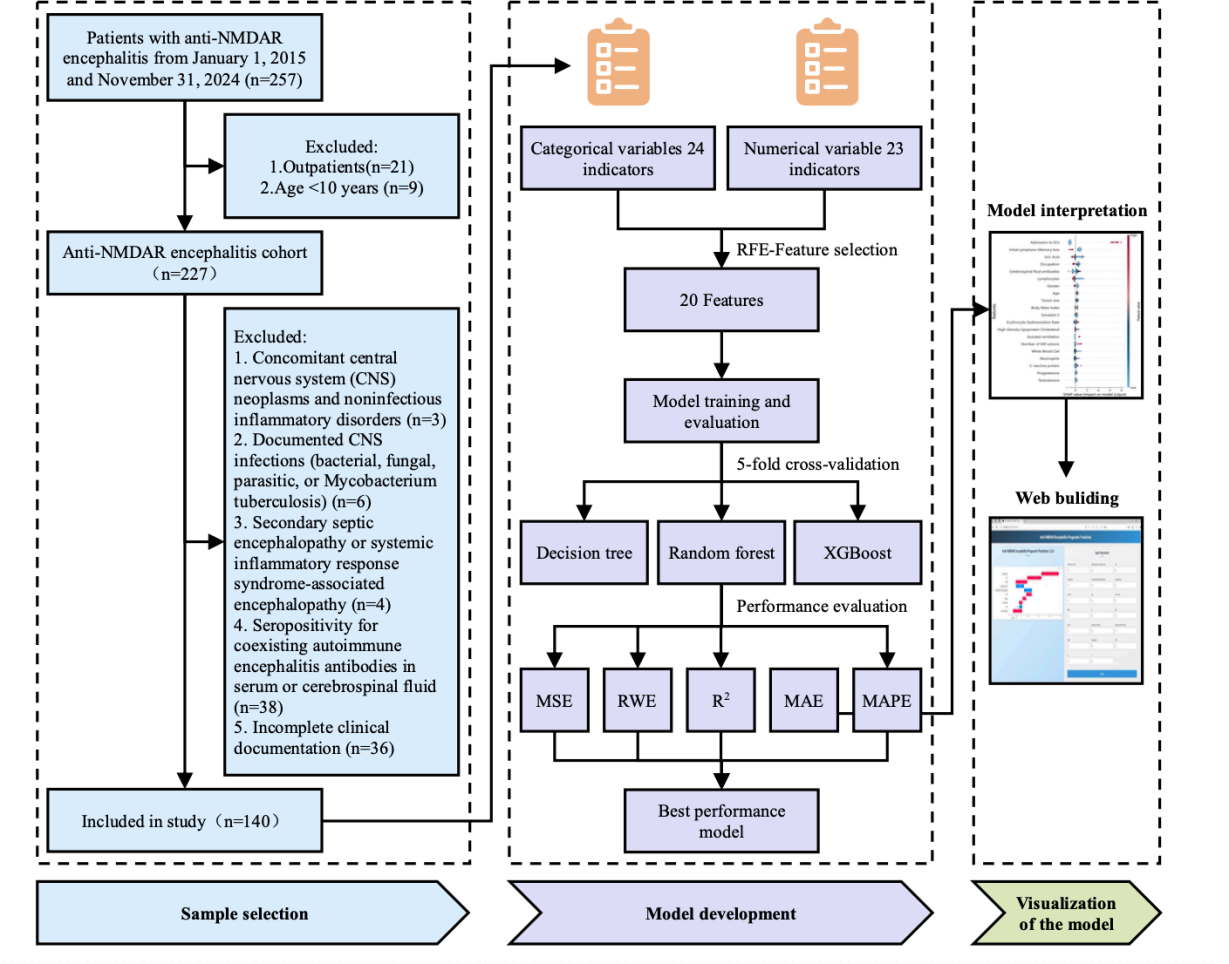
Flowchart of the study strategy. Anti-NMDAR: anti-N-methyl-D-aspartate receptor; MAE: mean absolute error; MAPE: mean absolute percentage error; MSE: mean square error; *R*²: coefficient of determination; XGBoost: extreme gradient boosting.

A retrospective cohort of 257 patients diagnosed with anti-NMDAR encephalitis was identified from the electronic medical records of the Third Affiliated Hospital of Sun Yat-sen University (January 2014-November 2024). According to the inclusion and exclusion criteria, 140 patients were included in the final analysis (Figure 1).

### Population Characteristics

**Figure 2.**
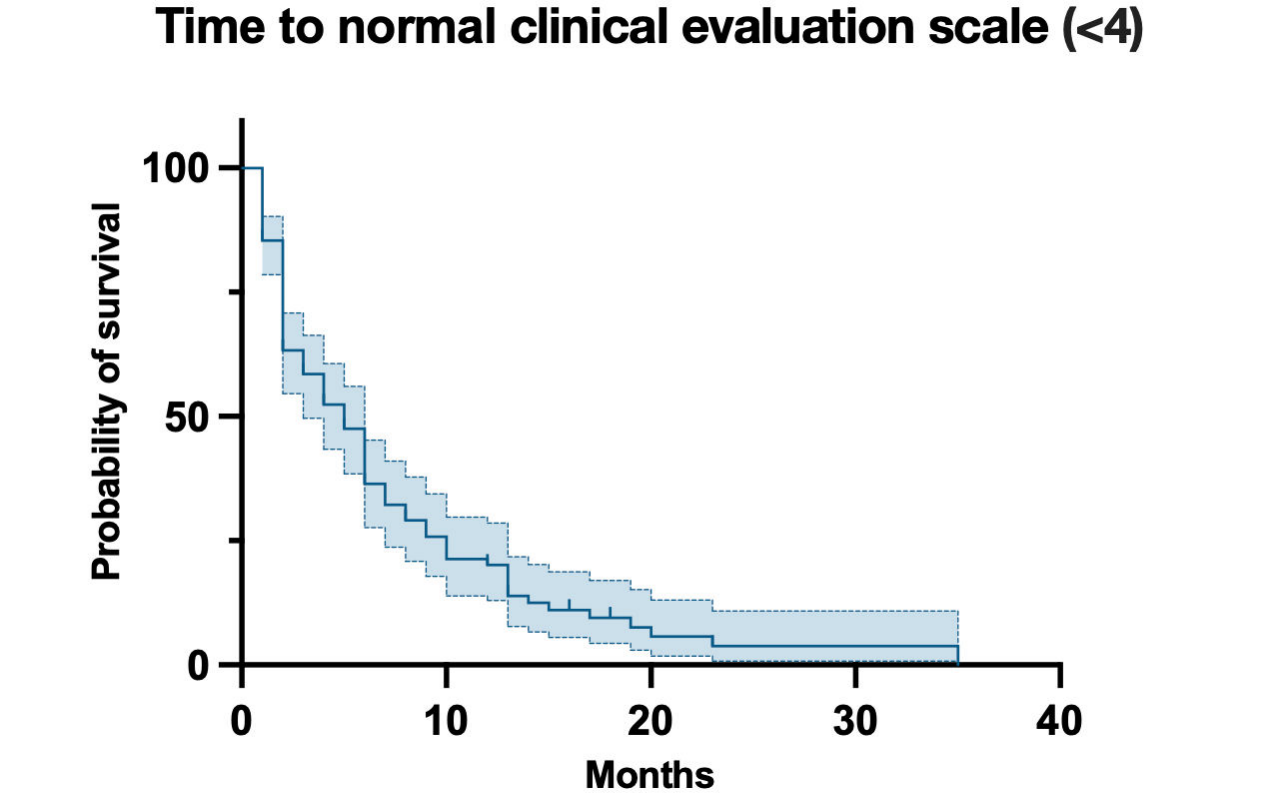
Recovery time of patients with anti-NMDAR encephalitis. NMDAR: anti-N-methyl-D-aspartate receptor.

Among the final cohort (median follow-up 8, IQR 2‐23 months), 102 of 140 (72.9%) were female patients, with a median age of 22 (IQR 17‐30) years; of these, 50 (35.7%) were students. Pulmonary infections emerged as the most frequent comorbidity, occurring in 54 of 140 (38.6%) patients, whereas fever was the leading prodromal symptom, appearing in 41 (29.3%) patients. NMDAR encephalitis–associated diffuse slow waves and delta brush patterns in electroencephalography were observed in 10% (14/140) and 2.1% (3/140) of cases, respectively. Among the 140 patients, 30 (21.4%) had tumors (28 benign teratomas and 2 malignancies). Median acute-phase CASE score was 11 (IQR 6‐16), improving to 0 (IQR 0‐1) at 1-year follow-up (Figure 2). Full demographic and clinical characteristics are detailed in Table 2.

**Table 2.** Clinical data baseline table of study subjects.

Predictive variables		Patients with NMDAR^a^
Age (years), median (IQR)		23 (18-31.8)
BMI (kg/m^2^), median (IQR)		21 (18-23.3)
Sex, n (%)		
Female		102 (72.9)
Male		38 (27.1)
Occupations, n (%)		
Student		50 (35.7)
Mental work		46 (32.9)
Manual labor		3 (2.1)
Others		41 (29.3)
Past medical history, n (%)		
None		126 (90)
Hypertension		1 (0.7)
Diabetes		3 (2.1)
Hepatitis B		4 (3)
Thyroid diseases		3 (2.1)
Tuberculosis		1 (0.7)
Syphilis		1 (0.7)
Tumors		1 (0.7)
Comorbidity, n (%)		
Pulmonary infection		54 (38.6)
Liver function damage		18 (12.9)
Thyroid lesions		11 (7.9)
Dysglycemia		15 (10.7)
Bleeding diseases		9 (6.4)
Respiratory failure		6 (4.3)
Urinary tract infections		6 (4.3)
Prodromal symptoms, n (%)		
Fever		41 (29.3)
Headache		29 (20.7)
Dizziness		5 (3.6)
Initial symptoms, n (%)		
Mental and behavioral abnormalities		99 (70.7)
Epilepsy		49 (35)
Disorders of consciousness		4 (2.9)
Memory loss		8 (5.7)
CSF^b^ indicators, median (IQR)		
WBC^c^		8 (2-28)
Protein		0.25 (0.17-0.35)
NMDAR antibodies		10 (10-32)
Blood indicators, median (IQR)		
NMDAR antibodies		10 (10-100)
Lymphocytes		1.35 (0.94-2.09)
Neutrophils		6.98 (4.75-9.88)
Monocytes		0.66 (0.43-0.86)
ESR^d^		14 (8-32)
CRP^e^		1.9 (0.6-5.3)
UA^f^		248 (152-335)
25-OHD^g^		44 (22.1-60)
IL-6^h^		5.9 (1.5-11.5)
HDL-C^i^		1.12 (0.89-1.33)
Testosterone		1.1 (0.74-1.6)
Follicle-stimulating hormone		3.51 (2.37-4.8)
Progestogen		0.7 (0.4-1.9)
Luteinizing hormone		2.89 (0.61-5.84)
Estradiol		96.5 (60.7-194)
ICU^j^ admission, n (%)		29 (20.7)
Mechanical ventilation, n (%)		26 (18.6)
EEG^k^, n (%)		
Normal		24 (17.1)
Mild abnormality		27 (19.3)
Moderate abnormality		19 (13.6)
Severe abnormality		13 (9.3)
Diffuse slow waves		14 (10)
Delta brush		3 (2.1)
Epileptic waves		10 (7.1)
Number of MR^l^ lesions, median (IQR)		0 (0-2)
Tumor markers, n (%)		
Negative		121 (86.4)
Slightly elevated		14 (10)
Significantly elevated		5 (3.6)
Combined with tumor, n (%)		
Yes		30 (21.4)
No		110 (78.6)
Number of tumors, n (%)		
1		28 (20)
2		2 (1.4)
Tumor size, median (IQR)		35.5 (19-53.5)
Duration of follow-up (months), median (IQR)		8 (2-23)

a NMDAR: anti-N-methyl-D-aspartate receptor.

b CSF: cerebrospinal fluid.

c WBC: white blood cell.

d ESR: erythrocyte sedimentation rate.

e CRP: C-reactive protein.

f UA: uric acid.

g 25-OHD: 25-hydroxyvitamin D.

h IL-6: Interleukin-6.

i HDL-C: high-density lipoprotein cholesterol.

j ICU: intensive care unit.

k EEG: electroencephalogram.

l MR: magnetic resonance.

### Variable Screening

This study examined predictors that longitudinally affect disease severity in the anti-NMDAR encephalitis observational cohort. The CASE score provides more insight into specific AE-related symptoms at onset, allowing for longitudinal assessment of these symptoms [17]. The predictor variables identified by RF were selected as common elements in the development of the prediction model. Select the importance threshold (*P*>.01) in the training set; these features were included in our study to construct a reduced feature set (selected features). A total of 20 of the 47 features were selected for inclusion in the ML model: 4 demographic characteristics (age, gender, BMI, and occupation), 3 clinical characteristics (admission to ICU, mechanical ventilation, initial symptoms-memory deficits), 11 laboratory parameters (UA, erythrocyte sedimentation rate, C-reactive protein, high-density lipoprotein cholesterol, CSF antibodies, neutrophils, estradiol, white blood cell, progestogen, and testosterone), and 2 neuroimaging characteristics (number of MR lesions and tumor size). The association between each candidate predictor is shown in Multimedia Appendix 1.

### Model Development and Performance Comparison

The prognostic performance of the selected features was systematically evaluated using 3 ML algorithms: RF, XGBoost, and DT. As detailed in Table 3, the RF model demonstrated superior discriminative capacity across all evaluation metrics (mean squared error=11.01, RMSE=3.32, *R*²=0.71, mean absolute error=2.49, and mean absolute percentage error=0.48). The RF model, selected as the optimal prognostic tool based on minimal RMSE, showed advantages in predicting functional outcomes of NMDAR encephalitis (the model parameters are shown in Table 4). A 5-fold cross-validation was used to verify the stability of the model (Table 1).

**Table 3.** Predictive performances of the 3 machine learning models.

Model	MSE^a^	RMSE^b^	*R*²^c^	MAE^d^	MAPE^e^
RF^f^	11.01	3.32	0.71	2.49	0.48
XGBoost^g^	14.98	3.87	0.61	2.87	0.53
DT^h^	26.88	5.18	0.29	3.92	0.59

a MSE: mean square error.

b RMSE: root-mean-squared error.

c R²: coefficient of determination.

d MAE: mean absolute error.

e MAPE: mean absolute percentage error.

f RF: random forest.

g XGBoost: extreme gradient boosting.

h DT: decision tree.

**Table 4.** Parameters of machine learning models.

Model and parameters		Values
RF^a^		
n_estimators		150
criterion		“gini”
max_depth		None
min_samples_split		2
min_samples_leaf		1
max_features		“sqrt”
bootstrap		True
random_state		42
XGBoost^b^		
n_estimators		150
learning_rate		0.1
max_depth		3
objective		“binary:logistic”
subsample		1.0
colsample_bytree		1.0
gamma		0
reg_alpha		0
reg_lambda		1
random_state		42
use_label_encoder		False
eval_metric		“logloss”
DT^c^		
criterion		“gini”
max_depth		None
min_samples_split		2
min_samples_leaf		1
max_features		None
random_state		42

a RF: random forest.

b XGBoost: extreme gradient boosting.

c DT: decision tree.

### Interpretation of the Model

**Figure 3.**
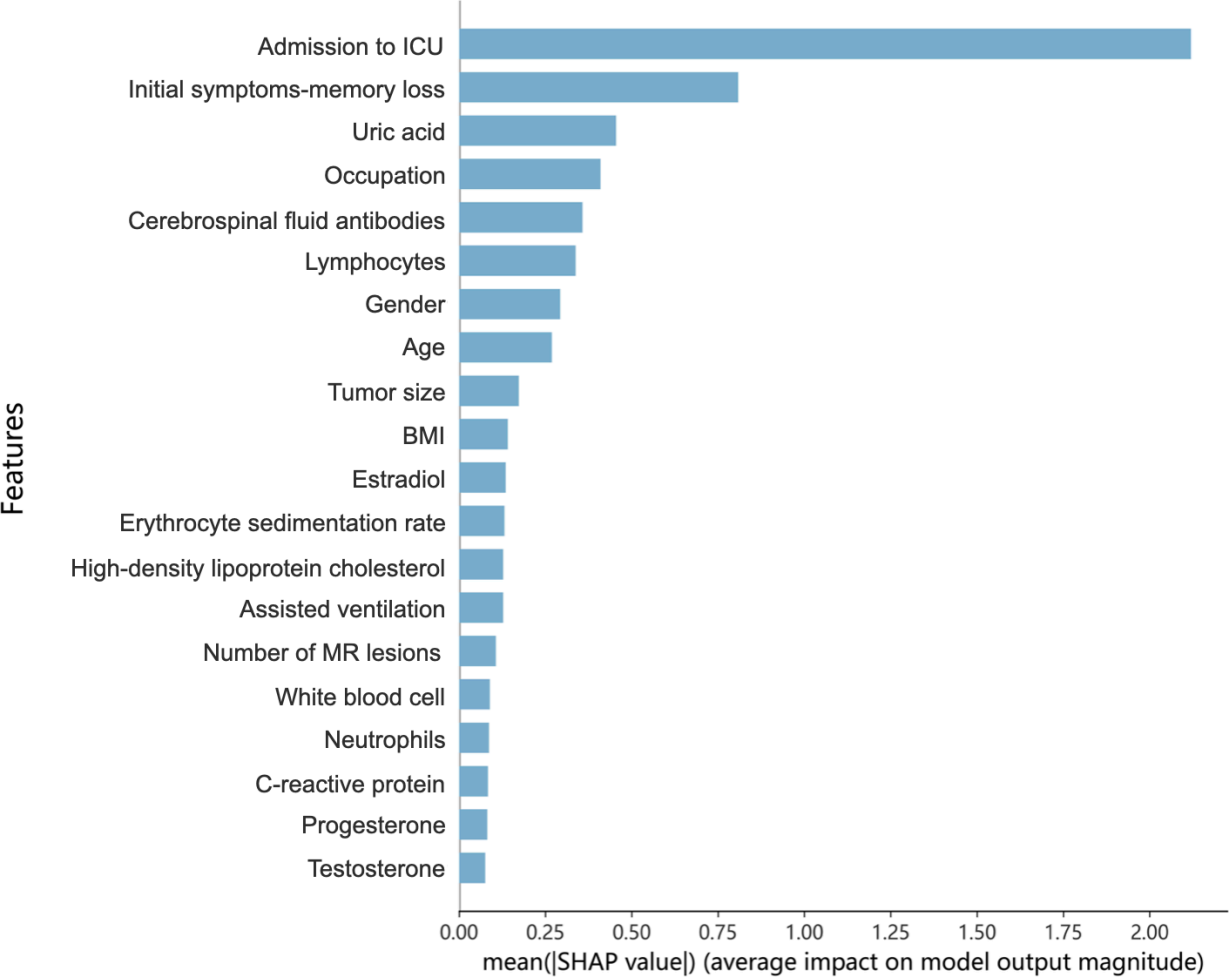
SHAP interpretation of the random forest model-importance score ranking of the model prediction characteristics. ICU: intensive care unit; RF: random forest.

To elucidate feature contributions and interpret model predictions, we used SHAP analysis, a robust game-theoretic approach that quantifies the relative importance of each predictive feature (Figures3  4). SHAP values represent the magnitude and directionality of each predictor’s influence on outcome probability, where positive values indicate increased disability risk. This visualization demonstrates how key features interact to determine individualized prognosis, enabling clinicians to prioritize modifiable risk factors. The summary plot (Figure 5) uses personalized feature attribution to represent range and distribution characteristics. We can directly see the impact of each feature on the prediction of prognostic features in NMDAR. Red to blue represents the eigenvalue from high to low. The thickness of the line represents the sample distribution.

**Figure 4.**
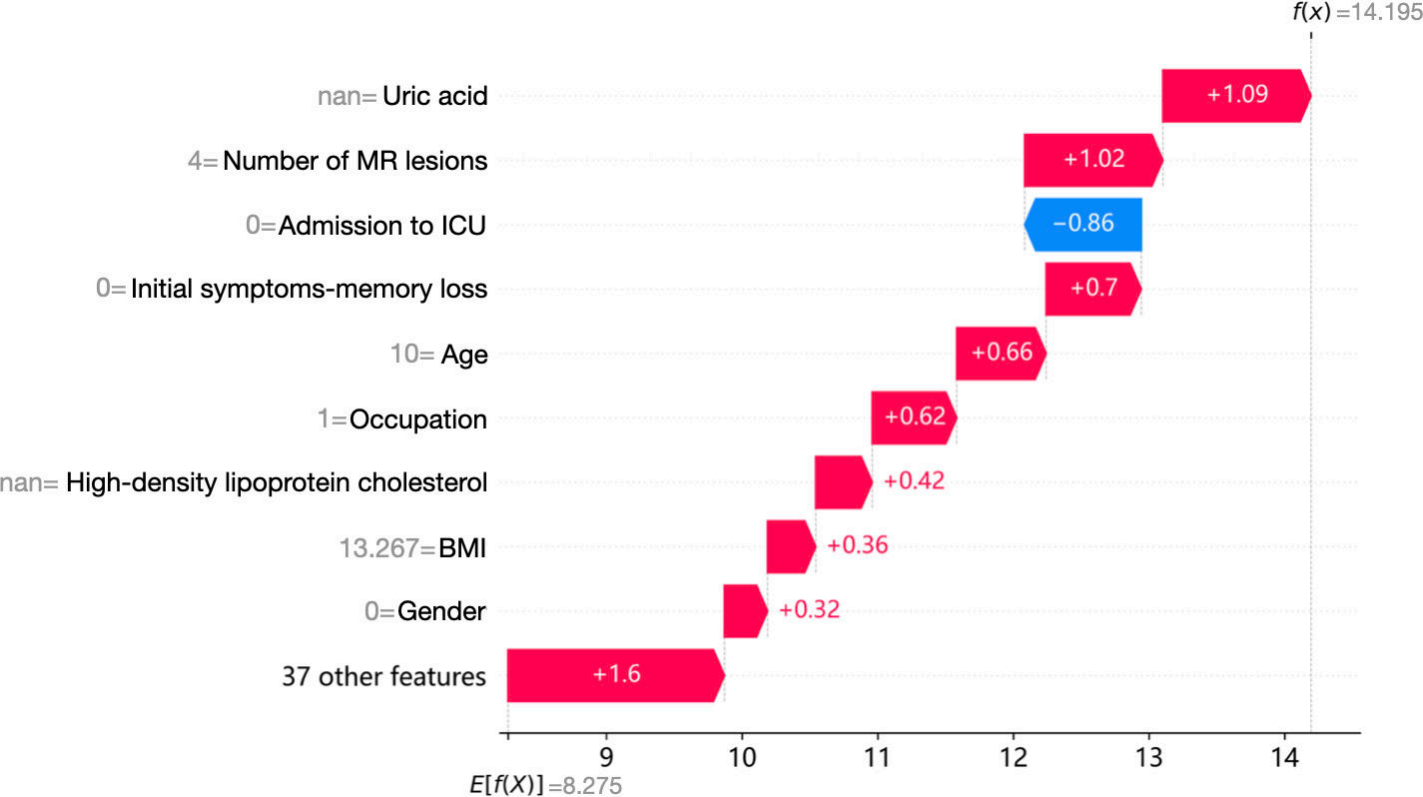
Shapley Additive Explanations interpretation of the random forest model-individualized prognostic impact assessment in anti-NMDAR encephalitis. ICU: intensive care unit; NMDAR: N-methyl-D-aspartate receptor; RF: random forest.

**Figure 5.**
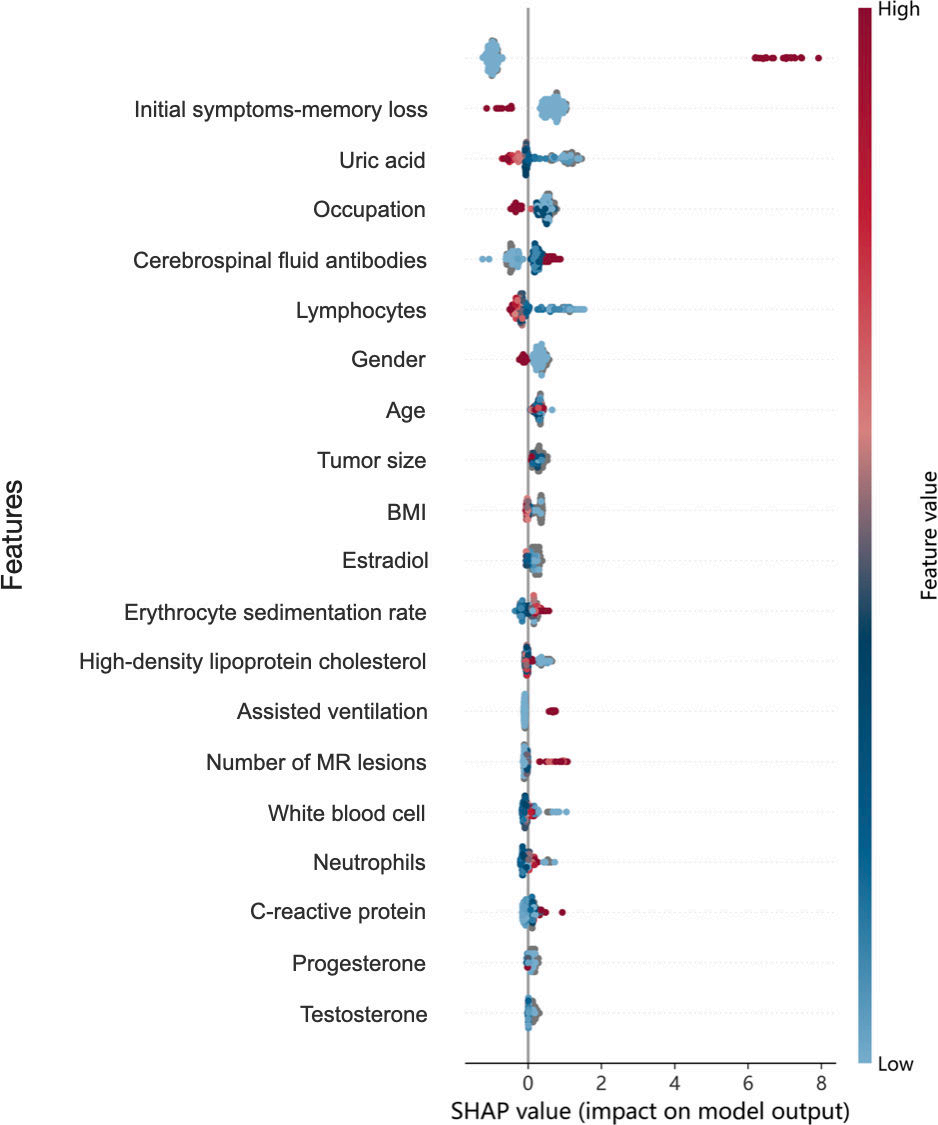
Shapley Additive Explanations summary plots of a 20-feature RF prediction model. ICU: intensive care unit; RF: random forest.

In the optimal performing RF model, the top 3 predictive features for prognostic predictors of NMDAR were admission to ICU (mean |SHAP value|=1.65), initial symptoms-memory loss (0.69), and UA (0.53). Other significant predictors included occupation, cerebrospinal fluid antibody titer, lymphocytes, sex, age, tumor size, BMI, estradiol, erythrocyte sedimentation rate, high-density lipoprotein cholesterol, need for assisted ventilation, number of MR lesions, white blood cell count, neutrophil count, C-reactive protein, progestogen, and testosterone.

### Development of Convenient Applications

**Figure 6.**
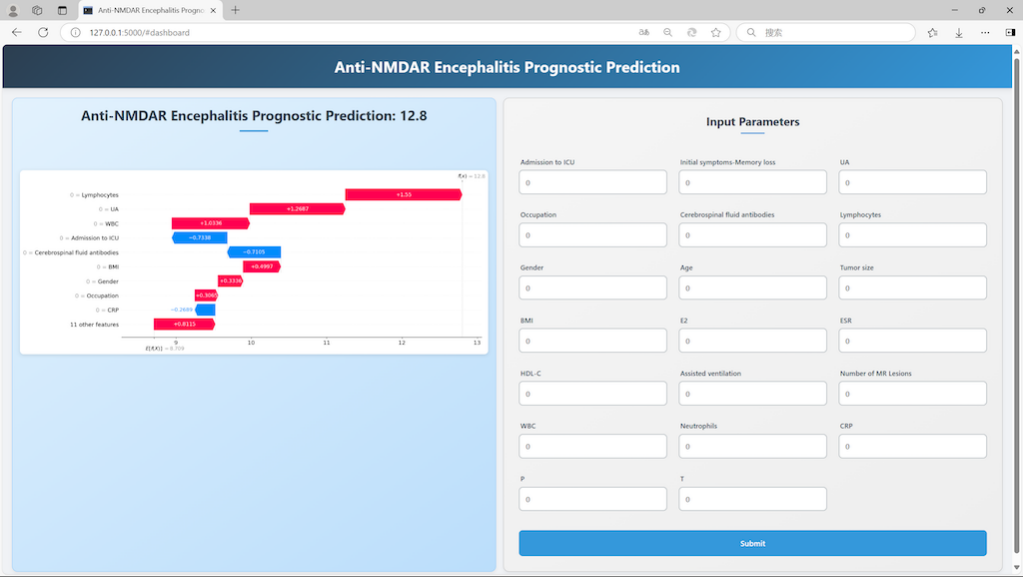
The application will automatically estimate the prognosis of anti–N-methyl-D-aspartate receptor encephalitis. NMDAR: N-methyl-D-aspartate receptor.

This platform features an intuitive graphical interface that accepts clinical input of 20 validated predictive variables, subsequently performing automated calculation of composite prognostic scores for patients with anti-NMDAR encephalitis through embedded algorithmic processing. Real-time risk stratification results provide clinicians with a quantitative prognostic assessment to support evidence-based treatment decisions. This decision support tool optimizes the process of translating predictive analytics into clinical practice (Figure 6).

## Discussion

### Principal Findings

We developed 3 ML-based prognostic models (XGBoost, RF, and DT) for anti-NMDAR encephalitis. A total of 20 pivotal predictors spanning demographic, clinical manifestations, laboratory parameters, and neuroimaging characteristics were identified from 47 candidate variables. The RF model exhibited the best performance. The SHAP interpretability analysis revealed that ICU admission (mean |SHAP value|=1.65), memory deficits at onset (0.69), and UA (0.53) were the most important predictive factors. To our knowledge, this is the first predictive model for anti-NMDAR encephalitis prognosis developed using ML.

Anti-NMDAR encephalitis is an autoimmune disease associated with antibodies targeting the GluN1 subunit of the NMDAR. Although it is the most common AE in adults, it is still considered a rare disease. Due to the widespread geographic distribution of patients, phenotypic heterogeneity, and insufficient understanding of the pathophysiological mechanisms, there are still many challenges in the diagnosis and treatment of this disease [18]. The outcomes for patients with anti-NMDAR encephalitis vary widely, with approximately 75% of patients achieving complete recovery or having only mild sequelae, while the remaining 25% experience severe neurological deficits or death [5,19]. Identifying patients at risk of severe disease progression and persistent deficits at an early stage is of great clinical significance, as it can inform individualized therapeutic decisions and facilitate timely escalation of treatment. Currently, there is a lack of quantitative predictive models for assessing disease prognosis.

In recent years, various ML algorithms have been widely applied in the field of medicine for treatment and prognosis prediction. Compared with traditional statistical methods, such as logistic regression, ML can directly capture nonlinear and complex interactions. Algorithms such as RF and light gradient boosting machine can automatically identify the most important predictive features, reducing the subjectivity of manual feature selection.

Current prognostic tools face critical challenges: (1) they are focused on single-indicator predictions, and traditional statistical methods were used for analysis. For example, Kashyap et al [20] used the neutrophil-to-lymphocyte ratio (NLR) for prognostic prediction in pediatric anti-NMDAR encephalitis, while Lin et al [11] used serum neuron-specific enolase for prognostic prediction. Such single-indicator prediction methods have obvious limitations in a rare disease with diverse manifestations and complex disease courses. (2) They rely on modified Rankin Scale that inadequately captures cognitive or psychiatric dimensions [12,21]. The modified Rankin Scale is a disability scale developed for stroke clinical trials and largely focuses on motor dysfunction. Such disability scales are limited in capturing nonmotor disabilities in AE. (3) They rely on delayed assessments, such as the anti-NMDAR encephalitis one-year functional status score [22], which requires a 4-week observation period and therefore lacks timeliness in prognostic scoring. For patients with poor prognosis who need timely treatment escalation or combination therapies, this delay may result in missed early therapeutic windows. (4) They depend on complex biomarkers requiring specialized processing: Xiang et al [10] developed a fusion prediction model based on 4 MRI sequences (T1WI, T2WI, FLAIR, and DWI) for early prediction of functional outcomes in adult patients with anti-NMDAR encephalitis. However, these indicators are not easily obtainable and require specialized radiologists to extract data, which severely limits clinical translation and application and is not conducive to clinical implementation.

We used 47 clinical variables available in the electronic medical records of a large tertiary hospital. The selection of these 47 variables was based on the following criteria: first, the aim of this study was to develop a practical prediction application for clinical settings; therefore, we needed to choose patient indicators that are easily accessible to clinicians. Second, according to previous literature and meta-analysis results, some variables were confirmed to be significantly related to patient prognosis. We also made certain improvements. Previous analyses of the predictive value of clinical MRI were based on binary assessments of normal or abnormal MRI. In this study, we used the number of lesions in MRI for analysis, which is more accurate. In feature selection, RFE with RF was applied. Features exceeding the importance threshold (*P*>.01) were retained. Ultimately, 20 influential variables were selected to develop a novel ML model to assess the prognosis of anti-NMDAR encephalitis.

For the outcome metric, CASE [15] is a disease severity scale specifically designed for AE and can clearly display the clinical trajectory from the acute phase to the recovery phase, making it suitable for longitudinal assessment of patient outcomes during treatment. The CASE score has not yet been used in prognostic model construction. Using the CASE score as the prognostic outcome variable in our study makes the prognostic assessment of patients more scientific and comprehensive.

We chose RF, DT, and XGBoost to construct predictive models for anti-NMDA encephalitis prognosis and evaluated the distinguishing features of each model through benchmark testing (mean squared error, RMSE, *R*², mean absolute error, and mean absolute percentage error) to determine the most suitable model for prediction. Ultimately, we selected the best-performing RF model and further deployed it on a web platform.

In our study, SHAP visualization analysis confirmed that admission to the ICU (mean |SHAP value|=1.65), initial symptoms of memory deficits (0.69), and UA levels are the main predictive factors for prognosis. In addition, other clinical features such as the presence of memory deficits, tracheostomy, and CSF antibody titers were linked to poorer prognoses in anti-NMDAR encephalitis.

In a retrospective study of 382 patients with anti-NMDAR encephalitis, Balu et al [23] identified ICU admission as the most significant univariate predictor [23]. A previous systematic study also revealed associations between decreased consciousness, ICU admission, and poorer outcomes in anti-NMDAR encephalitis [7]. Conversely, the binary risk stratification by ICU admission underscores the need for intensive monitoring protocols at disease onset. This is consistent with our result. SHAP analysis indicated that serum UA concentration plays a crucial role in predicting anti-NMDAR encephalitis prognosis, aligning with the findings of Liu et al [11]. SHAP dependence analysis revealed that UA values >400 μmol/L sharply reduced CASE probability (SHAP<−1.0). As a central nervous system autoimmune inflammatory disease, the pathogenesis of anti-NMDAR encephalitis remains unclear but is known to involve inflammatory responses. UA reduces proinflammatory cytokine release and removes reactive oxygen species, protecting the body from oxidative stress [24]. UA has also been reported to exert neuroprotective effects in various neurological diseases, such as Alzheimer disease, Parkinson disease, and multiple sclerosis [25]. Thus, UA likely serves as a protective factor in the course of anti-NMDAR encephalitis.

Refractory patients may benefit from alternative drugs such as tocilizumab, bortezomib, or daratumumab [10,18]. Our novel model, developed through rigorous variable selection, development, validation, interpretation, and web deployment, demonstrates excellent predictive accuracy. A significant advantage is that all predictive factors used in the model are easily obtainable by clinicians, making risk stratification within 24 hours, enabling them to promptly identify patients with poor prognoses and guide more aggressive treatment escalation to prevent mild cases from progressing to treatment-resistant severe encephalitis.

### Strengths and Limitations

The strengths of this study lie in three advancements: (1) We developed a novel ML model for prognostic prediction in encephalitis, (2) this model is exclusively based on clinically accessible parameters, and (3) we created a visualized web-based decision support platform to enable real-time clinical implementation. Our study also has several limitations. First, due to the rarity of the disease, the sample size was relatively small, and it is impossible to conduct subgroup analysis and statistics for each clinical manifestation. Second, the data used for model development were obtained from patients admitted to a specific medical institution rather than from a large database, which may introduce bias. Future research should consider training and validating the model across different national contexts.

### Conclusions

In this study, we developed and validated an interpretable ML model based on the RF algorithm. The model demonstrated high predictive accuracy, with robust performance metrics, supporting its potential utility in clinical settings. Through SHAP analysis, key prognostic predictors were identified, including admission to ICU, memory deficits at initial presentation, and serum UA levels, offering novel insights into risk stratification. This tool may assist clinicians in making evidence-based decisions, optimizing interventions, and ultimately improving outcomes for patients with anti-NMDAR encephalitis.

## Supplementary material

10.2196/75020 Multimedia Appendix 1 Association between each candidate predictor.

## Abbreviations

AE: autoimmune encephalitis
Anti-NMDAR: anti-N-methyl-D-aspartate receptor
CASE: Clinical Evaluation Scale for Autoimmune Encephalitis
CSF: cerebrospinal fluid
DT: decision tree
ICU: intensive care unit
ML: machine learning
MRI: magnetic resonance imaging
MSE: mean squared error
NLR: neutrophil-to-lymphocyte ratio
RF: random forest
RFE: recursive feature elimination
RMSE: root-mean-squared error
SHAP: Shapley Additive Explanations
TRIPOD: Transparent Reporting of a Multivariable Prediction Model for Individual Prognosis or Diagnosis
UA: uric acid
XGBoost: extreme gradient boosting
