# Supplementary figures and images for: Machine Learning and Shapley Additive Explanations Value Integration for Predicting the Prognostic of Anti-N-Methyl-D-Aspartate Receptor Encephalitis: Model Development and Evaluation Study
Source: JMIR Med Inform. 2025 Sep 22;13:e75020. doi: 10.2196/75020 (PMC12453450; doi:10.2196/75020)

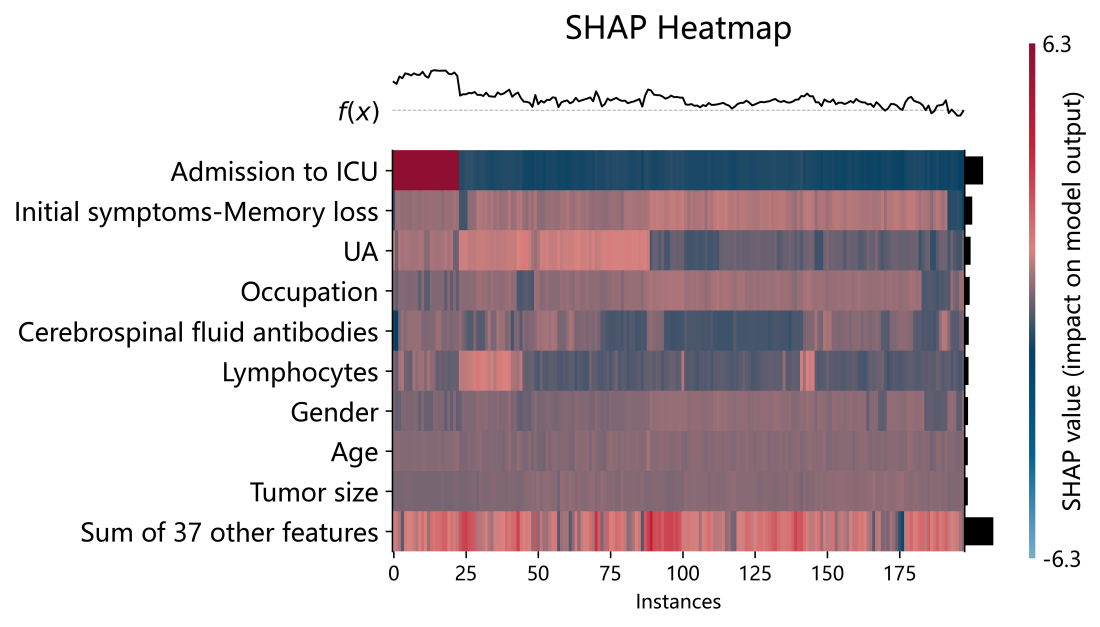

Supplement: Multimedia Appendix 1 [file medinform-v13-e75020-s001.png]
